# Practical Considerations on the Multi-layer Mirrors for Superluminal Ring Laser Gyroscopes

**DOI:** 10.1038/srep12026

**Published:** 2015-07-09

**Authors:** Zhiguo Wang, Baolun Yuan

**Affiliations:** 1 College of Opto-Electronic Science and Engineering, National University of Defense Technology, Changsha 410073, China; 2 Interdisciplinary Center of Quantum Information, National University of Defense Technology, Changsha 410073, China

## Abstract

We have created a simple model to analyze the restrictions on superluminal ring laser gyroscopes arising from the absorption of coating materials. For a ring laser gyroscope with a cavity length of 15 cm, the scale factor enhancement is nearly impossible due to absorption from the high dispersion mirror. In order to obtain a practical superluminal ring laser gyroscope, the extinction coefficient of coating materials should be less than 1E-10, which is a challenge at present.

Superluminal effects have many potential applications, ranging from optical data buffers to sensitivity-enhanced interferometers^1^,^2^,^3^. One important application is the superluminal ring laser gyroscope^4^,^5^, which may enable purely self-contained navigation. It is also possible to detect the gravitational frame-dragging effect by measuring the Lense-Thirring rotation on the Earth^6^. Recently, several methods were proposed to enhance the sensitivity of ring laser gyroscopes with superluminal effects, such as alkali metal vapor cells^7^, coupled optical resonators^8^, and using multilayer optical coatings on the reflection mirror^9^. The latter is very attractive due to its compact configuration, vibration-robust mirror, and broad bandwidth. However, strong dispersion systems are always accompanied by intense absorption. Therefore, the loss associated with dispersion should be considered before we could obtain a practical mirror for superluminal ring laser gyroscopes. In this short communication, we analyzed the restrictions of absorption losses in the multilayer coatings for a superluminal ring laser gyroscope. We found that the enhancement factor is greatly reduced due to coating losses, and a practical superluminal ring laser gyroscope is hard to be obtained with any current coating technology.

## Results

Even the best coating material has absorption, which is usually characterized by an extinction coefficient *κ*, or the absorption coefficient^10^,


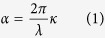


where *λ* is the wavelength, and *κ* wavelength-dependent.

Both theory and experiments have shown that absorption loss of dispersive dielectric mirrors is proportional to the group delay for high reflectance mirrors^11^. The rigid analysis can be obtained by calculating the power reflectivity of multi-layer mirrors with the transfer matrix method which is a very fundamental tool in thin film coating related books^10^. However, it is sufficient to use a simpler estimation method as described in^12^,^13^. If we denote the group delay time of the multi-layer coating for reflection as *τ*_*g*_, the corresponding optical length is


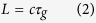


where *c* is the speed of light in vacuum. The absorption loss can be written as


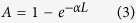


For high reflection mirrors, the absorption loss is very small, so we can approximate this as


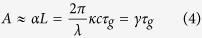


where *γ* stands for the proportionality coefficient between the absorption loss and the group delay time^13^. With *λ *= 800 nm, *κ* = 10^−4^, *c* = 3 × 10^8^m/s, we obtain *γ* *=* 2.355 × 10^11^ per second, which is roughly the same as the results from^12^,^13^. In^14^, analytic expressions are given for the reflection delay, penetration depth and absorption of a quarter wave mirror based on energy conservation considerations, which agrees with equation (4).

It is obvious that reducing the extinction coefficient *κ* could reduce absorption. However, even for the best coating materials and coating technologies, the extinction coefficient is still roughly *κ* *=* 10^−6^ 15. The extinction coefficients of the usual coating materials are roughly 10^–3^ ~ 10^–4^ 16,17. We assume the coating materials and technology are the best currently available, so we use *γ* *=* 2.355 × 10^9^ for an estimate, which corresponds to *κ* *=* 10^–6^. A typical ring laser has a cavity length of about 15 cm, so the group delay time should be approximately 0.5 ns in order to obtain a good superluminal ring laser gyroscope. In this case, the absorption calculated using equation (4) is larger than 1, which makes it impossible to realize a laser. It should be noted that if the absorption is too large, the estimation method used above is not effective and rigid numerical calculations should be used.

From another perspective, the total loss of a typical mirror is less than 10^–4^ in a practical laser gyroscope^18^. This restricts the group delay time to 0.42 × 10^–13^ s. As such, the enhanced scale factor for a 15 cm-length ring laser gyroscope is^9^,


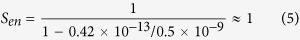


This means we cannot obtain an effective scale factor enhancement.

On the other hand, if we want to use a mirror with *τ*_*g*_ = 0.42 × 10^–13^ s to obtain a scale factor enhancement of 2, the cavity length of the ring laser gyroscope should be no larger than 0.0276 mm, which is impossible to realize, because the cavity length is roughly the same size as the coating thickness.

## Discussion

The idea of a superluminal ring laser gyroscope with the multi-layer coating mirror is very attractive, but the absorption of the coating makes it difficult to produce with current technologies. If the extinction coefficient of the coating material could be reduced to ~10^–10^, this kind of ring laser gyroscope may be possible. However, other factors such as scattering would also need to be considered.

In conclusion, we analyzed the restrictions to the potential superluminal ring laser gyroscope with multi-layer coating mirrors due to coating absorption. It was found that in order to obtain a practical superluminal ring laser gyroscope, the extinction coefficient of the coating material should be less than 10^–10^, which is a challenge at present, since even the bulk materials have an extinction coefficient of roughly 10^–10^ 15.

## Methods

The absorption of multilayer coatings is calculated according to a simple analytic model as shown in the text, which is cited from several references. The enhanced scale factor is calculated based on formulas in^9^.

## Additional Information

**How to cite this article**: Wang, Z. & Yuan, B. Practical Considerations on the Multi-layer Mirrors for Superluminal Ring Laser Gyroscopes. *Sci. Rep.* **5**, 12026; doi: 10.1038/srep12026 (2015).
